# Correlation between mass transfer coefficient k_L_a and relevant operating parameters in cylindrical disposable shaken bioreactors on a bench-to-pilot scale

**DOI:** 10.1186/1754-1611-7-28

**Published:** 2013-12-02

**Authors:** Wolf Klöckner, Riad Gacem, Tibor Anderlei, Nicole Raven, Stefan Schillberg, Clemens Lattermann, Jochen Büchs

**Affiliations:** 1 AVT. Biochemical Engineering, RWTH Aachen University, Worringerweg 1, Aachen 52074, Germany; 2 Adolf Kühner AG, Dinkelbergstrasse 1, Birsfelden 4127, Switzerland; 3 Fraunhofer Institute for Molecular Biology and Applied Ecology (IME), Forckenbeckstrasse 6, Aachen 52074, Germany

**Keywords:** Dimensional analysis, Maximum oxygen transfer capacity, Orbitally shaken, Plant cell suspension cultures, Single-use, Volumetric mass transfer coefficient

## Abstract

**Background:**

Among disposable bioreactor systems, cylindrical orbitally shaken bioreactors show important advantages. They provide a well-defined hydrodynamic flow combined with excellent mixing and oxygen transfer for mammalian and plant cell cultivations. Since there is no known universal correlation between the volumetric mass transfer coefficient for oxygen k_L_a and relevant operating parameters in such bioreactor systems, the aim of this current study is to experimentally determine a universal k_L_a correlation.

**Results:**

A Respiration Activity Monitoring System (RAMOS) was used to measure k_L_a values in cylindrical disposable shaken bioreactors and Buckingham’s π-Theorem was applied to define a dimensionless equation for k_L_a. In this way, a scale- and volume-independent k_L_a correlation was developed and validated in bioreactors with volumes from 2 L to 200 L. The final correlation was used to calculate cultivation parameters at different scales to allow a sufficient oxygen supply of tobacco BY-2 cell suspension cultures.

**Conclusion:**

The resulting equation can be universally applied to calculate the mass transfer coefficient for any of seven relevant cultivation parameters such as the reactor diameter, the shaking frequency, the filling volume, the viscosity, the oxygen diffusion coefficient, the gravitational acceleration or the shaking diameter within an accuracy range of +/− 30%. To our knowledge, this is the first k_L_a correlation that has been defined and validated for the cited bioreactor system on a bench-to-pilot scale.

## Background

The success of new biopharmaceuticals highly depends on their potential to compete with established products. Parameters such as a fast time to market, cost effectiveness and manufacturing flexibility are key issues that need to be considered while maintaining product quality [1]. Using disposable equipment can help reduce investment costs and increase flexibility and process safety [2] ultimately leading to enhanced competitiveness of the products. Consequently, disposable bioreactors are increasingly being used in biotechnological production processes within the past ten years [3].

Today, one of the most popular disposable bioreactors is the WAVE cultivation system that was first described in 1976 [4,5]. Since then, various other types of disposable cultivation systems for different applications have been developed. A wide range of different reactor sizes, starting with disposable screening systems in microliter scale, up to bioreactors with capacities of several cubic meters are on the market [3]. Available cultivation systems and their applications are described in several review articles [2,3,6,7].

Despite the increasing usage of disposable cultivation systems, only few scientific publications have reported about their characterization with respect to power input, oxygen transfer and mixing performance [8]. By contrast, stirred stainless steel bioreactors have been extensively characterized and optimized over the past 60 years [3]. A direct design transfer from stainless steel to stirred disposable bioreactor systems is not feasible because of different properties of the applied materials. For instance, it is not possible to keep geometric similarity between a stirrer made of stainless steel and a disposable stirrer, due to the reduced strength and rigidity of the disposable polymer material. New agitation concepts for disposable bioreactors are desired to simplify their design and make them more cost-efficient [6]. Surface aerated reactors without complex built-in components fulfil the requirement for a cost-efficient reactor design [9]. In these systems, oxygen transfer and power input are either introduced by a wave, rocking or shaking motion of the bioreactor [3]. Orbitally shaken bioreactors are advantageous due to their well-defined liquid distribution that allows a precise characterization of power input and oxygen transfer. The magnitude of volumetric power input in these systems is comparable to that of conventional stirred tank reactors [10,11], indicating excellent mixing characteristics also for liquids at elevated viscosity.

In the 50 mL scale, the orbitally shaken TubeSpin Bioreactor was developed and tested as a high throughput system for mammalian cell cultivation [12,13]. On a larger scale, different studies reported about power input [10,11,14,15], mixing properties [16] and scale-up performance [15,17-20] of disposable orbitally shaken reactors. In several studies the volumetric mass transfer coefficient k_L_a was identified as one of the main parameters for successful scale-up and transfer of cultivation conditions from stirred tank to disposable shaken reactors [13,18,21,22]. Zhang et al. [23] also described a helical track attached on the inner reactor wall as a potential means to increase the mass transfer coefficient. However, a scale- and volume-independent k_L_a correlation for cylindrical orbitally shaken bioreactors has not been described so far. Thus, the aim of this current study is to experimentally determine such a k_L_a correlation in cylindrical disposable shaken bioreactors with volumes from 2 L up to 200 L and apply it to the cultivation of suspended plant cells.

## Results and discussion

### Dimensional analysis

Dimensional analysis according to Buckingham’s π -Theorem was used to define a scale- and volume-independent k_L_a correlation [24,25]. According to the rules of Buckingham’s theory, the resulting correlation is restricted to systems with geometric similarity. Influencing variables for k_L_a in cylindrical orbitally shaken bioreactors and their corresponding units are the volumetric mass transfer coefficient for oxygen $k_{L}a\;\left[\frac{1}{s}\right]$, the reactor diameter d [m], the shaking diameter d_0_ [m], the shaking frequency $n\;\left[\frac{1}{s}\right]$, the liquid volume V_L_ [m^3^], the diffusion coefficient for oxygen $D_{O2}\;\left[\frac{m^{2}}{S}\right]$, the kinematic viscosity $\nu \;\left[\frac{m^{2}}{s}\right]$ and the gravitational acceleration $g\;\left[\frac{m}{s^{2}}\right]$. The following dimensionless numbers were formed with the influencing variables according to the rules of Buckingham’s theory:

Mass transfer number 

$$k_{L}a\cdot {\left(\frac{\nu }{g^{2}}\right)}^{\frac{1}{3}}$$

Froude number 

$$\frac{n^{2}\cdot d_{0}}{g}$$

Volume number 

$$\frac{V_{L}}{d^{3}}$$

Geometric number 

$$\frac{d_{0}}{d}$$

Galilei number 

$$\frac{d^{3}\cdot g}{\nu^{2}}$$

Schmidt number 

$$\frac{\nu }{D_{O2}}$$

This set of dimensionless numbers was also proposed by Henzler and Schedel [26] to develop a scale-independent correlation for the mass transfer coefficient in shake flasks. The applied numbers for surface aerated bioreactors differ from dimensionless numbers that are commonly used to describe the mass transfer in bubble aerated bioreactors because the principle of mass transfer differs fundamentally in both systems. In contrast to surface aerated orbitally shaken bioreactors, mass transfer in bubble aerated bioreactors depends on the amount, size, break-up and coalescence of gas bubbles. The characteristics of gas bubbles are strongly influenced by the volumetric power input, which is therefore commonly used as a parameter for k_L_a correlations for bubble aerated bioreactors. These aspects are not relevant for the utilized surface aerated bioreactors that are operated without bubble aeration.

The numbers are independent from one another and the set of numbers can be transformed into a different set of dimensionless numbers, with no influence on the resulting exponents of the included variables, as long as the rules of Buckingham’s π -Theorem are preserved [25]. In this set of dimensionless numbers the Froude number is used to express the ratio of centrifugal to gravitational forces during shaking. The relative filling volume is considered with the Volume number and the Geometric number is used to describe the ratio of shaking diameter to reactor diameter. The height and shape of the free liquid surface during shaking is influenced by the ratio of gravitational to viscous forces, expressed by the Galilei number. The ratio of momentum transfer to diffusive mass transfer in the gas/liquid surface is described by the Schmidt number. In bioreactors without bubble aeration, the influence of the surface tension on the mass transfer coefficient decreases with increasing reactor size as already described by Doig et al. [27] and Hermann et al. [28] for microtiter plates. Consequently, the influence of the surface tension was not considered in orbitally shaken vessels with volumes from 2 L to 200 L. The occurrence of gas bubbles was avoided by using surface aeration only and the smooth surface of the inner reactor wall led to a well-defined and homogeneous liquid distribution during shaking. The following power law function was used to describe the correlation among the dimensionless numbers:

(1) $$k_{L}a\cdot {\left(\frac{\nu }{g^{2}}\right)}^{\frac{1}{3}}=C\cdot {\left(\frac{n^{2}\cdot d_{0}}{g}\right)}^{\alpha }\cdot {\left(\frac{V_{L}}{d^{3}}\right)}^{\beta }\cdot {\left(\frac{d_{0}}{d}\right)}^{\gamma }\cdot {\left(\frac{d^{3}\cdot g}{\nu^{2}}\right)}^{\delta }\cdot {\left(\frac{\nu }{D_{O2}}\right)}^{\epsilon }$$

Each exponent in Eq. 1 has to be determined separately by empirically varying the corresponding dimensionless number.

### Influence of the critical circulation frequency

As described by several authors, a critical circulation frequency N_c_ has to be exceeded to induce a rotating liquid motion in cylindrical orbitally shaken vessels [10,29]. Hardly any movement was observed between liquid bulk and reactor wall below the critical circulation frequency, resulting in very low power input and oxygen transfer values. Consequently, only k_L_a values that were measured at shaking frequencies above the critical circulation frequency were considered for determining the k_L_a correlation. The following equation was used to calculate the critical circulation frequency [10]:

(2) $$N_{c}=\frac{1}{d^{2}}\cdot \sqrt{0.28\cdot V_{L}\cdot g}$$

According to Eq. 2, the critical circulating frequency N_c_ is a function of the inner reactor diameter d, the filling volume V_L_ and the acceleration of gravity g.

### Out-of-phase operation in orbitally shaken bioreactors

The transition from in-phase to out-of-phase operation in orbitally shaken bioreactors is accompanied by a strong decrease in power input and oxygen transfer [30-32]. Liquids with high viscosities in systems with low shaking diameters are prone to out-of-phase operation as described in several studies for shake flasks [32,33]. Thus, a minimum value for the ratio of shaking diameter (d_0_) to reactor diameter (d), expressed by the Geometric number, is required to avoid an undesired out-of-phase operation in cylindrical orbitally shaken bioreactors.

### Defining the k_L_a correlation

**Figure 1:**
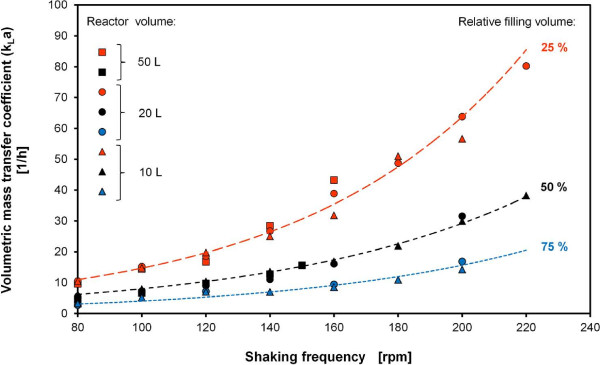
**k**_**L**_**a values of 10 L, 20 L and 50 L bioreactors at 25°C determined with RAMOS using 1 M Na**_**2**_**SO**_**3**_**solution with 10**^**-7**^**M CoSO**_**4**_**, 0.012 M phosphate buffer, initial pH = 8, data points connected with trend lines for relative filling volumes of 25%, 50% and 75%.**

Values for k_L_a in cylindrical bioreactors with nominal volumes from 2 L to 50 L were determined according to Eq. 11 by using a RAMOS device in combination with a 0.5 M or 1 M sulfite system for OTR_max_ measurements. Values for k_L_a in cylindrical reactors of different size are presented in Figure 1. A decrease in k_L_a with increasing filling volume was observed. However, similar k_L_a values were obtained for the same relative filling volume in the applied scales (10 L - 50 L). This result is, on the first glance, astonishing as the volumetric transfer area (a) decreases with increasing vessel size. Similar k_L_a values in different scales for the same relative filling volume can be explained by an increasing value for the coefficient k_L_ with increasing reactor size because of a higher power input [10]. A precise characterization of the mass transfer coefficient using dimensional analysis is presented in the following section.

**Figure 2:**
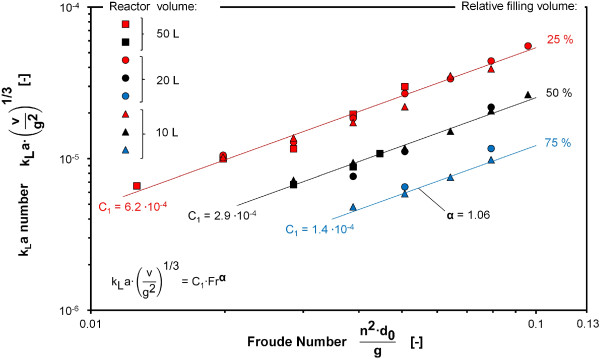
**k**_**L**_**a number as a function of the Froude number for 10 L, 20 L and 50 L bioreactors at 25°C, k**_**L**_**a values determined with RAMOS using 1 M Na**_**2**_**SO**_**3 **_**solution with 10**^**-7**^**M CoSO**_**4**_**, 0.012 M phosphate buffer, initial pH = 8, power functions with average exponent α = 1.06 fitted to values for relative filling volumes of 25%, 50% and 75% at shaking frequencies of 80–220 rpm with n < N**_**C**_**according to Eq.**2**.**

The influence of the Froude number on the k_L_a number was determined by varying the shaking frequency (n) as shown in Figure 2. A variation of the shaking frequency only affects the Froude number while all remaining dimensionless numbers are kept constant, allowing the influence of the Froude number to be separated from the influence of the other dimensionless numbers. An average exponent of α = 1.06 was determined as shown in Figure 2. The validity of the exponent α is restricted to a range for the Froude number of between 0.013 and 0.097 as depicted in Figure 2.

**Figure 3:**
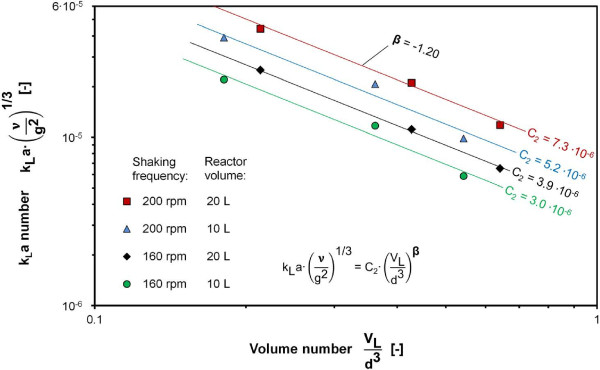
**k**_**L**_**a number as a function of the Volume number for 10 and 20 L bioreactors at 25°C, relative filling volume varied between 25% and 75% at shaking frequencies of 160 rpm and 200 rpm, k**_**L**_**a values determined with RAMOS using 1 M Na**_**2**_**SO**_**3 **_**solution with 10**^**-7**^**M CoSO**_**4**_**, 0.012 M phosphate buffer, initial pH = 8, power functions with average exponent β = −1.20 fitted to data points.**

The influence of the Volume number in Eq. 1 was determined by varying the filling volume, which only affects the Volume number. Thus, the resulting average exponent can be directly specified from the measuring values presented in Figure 3. An average exponent of β = -1.20 was determined for the influence of the Volume number. The range of applicability for the exponent β is restricted to values of the Volume number of between 0.18 and 0.65.

**Figure 4:**
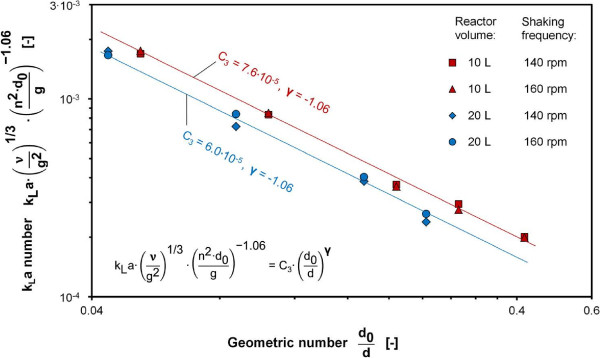
**Product of k**_**L**_**a number and Froude number as a function of the Geometric number for 10 L and 20 L bioreactors at 25°C, shaking diameter d**_**0 **_**varied between 1.25 cm and 10 cm at shaking frequencies of 140 rpm and 160 rpm, k**_**L**_**a values determined with RAMOS using 1 M Na**_**2**_**SO**_**3 **_**solution with 10**^**-7**^**M CoSO**_**4**_**, 0.012 M phosphate buffer, initial pH = 8, power functions with average exponent γ = -1.06 fitted to data points.**

The influence of the Geometric number on the k_L_a number was investigated by altering the shaking diameter d_0_. A modification of the shaking diameter in Eq. 1 leads to a variation of the Geometric number and the Froude number. Therefore, the influence of the Froude number on the k_L_a number had to be considered with the exponent α = 1.06. This calculation was conducted by multiplying the k_L_a number with the inverse Froude number as stated in Figure 4. An average exponent of γ = -1.06 was determined within a range of variation for the Geometric number from 0.04 to 0.42 as shown in Figure 4. A low value for the ratio of shaking diameter (d_o_) to reactor diameter (d), which is expressed with the Geometric number, can lead to an undesired out-of-phase operation. Thus, the minimum value for the Geometric number was set to 0.06 to prevent an out-of-phase operation during the measurements in the present work. The final range of applicability for the exponent γ of the Geometric number was defined between 0.06 and 0.42.

**Figure 5:**
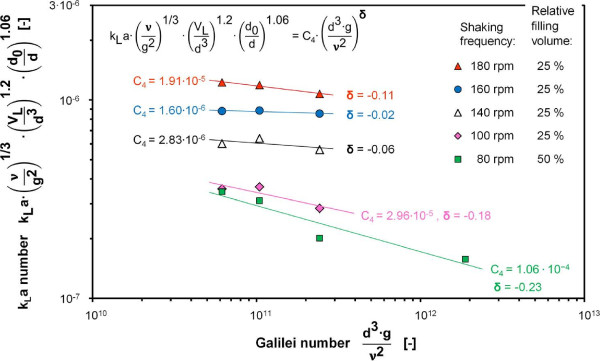
**Product of k**_**L**_**a number, Volume number and Geometric number as a function of the Galilei number measured at 25°C, reactor diameter varied between 0.25 cm and 0.75 cm using 10 L, 20 L, 50 L and 200 L bioreactors, k**_**L**_**a values determined with RAMOS using 1 M Na**_**2**_**SO**_**3 **_**solution with 10**^**-7**^**M CoSO**_**4**_**, 0.012 M phosphate buffer, initial pH = 8, power functions fitted to data points for different shaking frequencies and relative filling volumes, average exponent δ = -0.12.**

Vessels of different sizes were used to vary the reactor diameter and, in this way, the Galilei number. The influence of the Volume number and the Geometric number were considered with their respective exponents and multiplied with the k_L_a number as stated in Figure 5. An average exponent of δ = -0.12 was determined with a validity range for the Galilei number of between 6.2∙10^10^ and 1.88∙10^12^.

**Figure 6:**
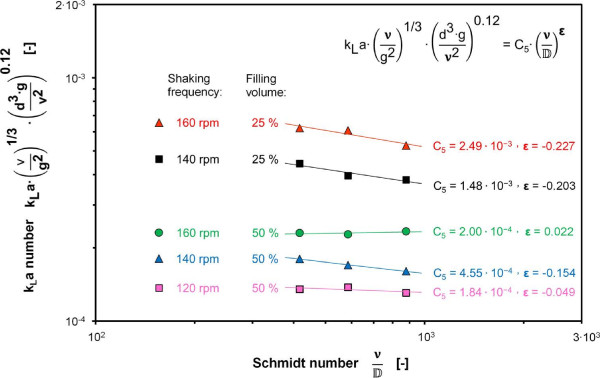
**Product of k**_**L**_**a number and Galilei number as a function of the Schmidt number (Sc) measured with 20 L bioreactors at 25°C using water (Sc = 416) and sulfite solutions with 0.5 M (Sc = 580) and 1 M Na**_**2**_**SO**_**3 **_**(Sc = 877) with 10**^**-7**^**M CoSO**_**4**_** and 0.012 M phosphate buffer, initial pH = 8, power functions fitted to data points for different shaking frequencies and filling volumes, determined average exponent ϵ = -0.12.**

The variation of the Schmidt number in Eq. 1 was realized by varying the diffusion coefficient for oxygen $\left(D_{O2}\right)$. Sodium sulfite (Na_2_SO_3_) solutions with concentrations of 0.5 mol/L and 1 mol/L and measurements with deionized water, using the dynamic gassing-out method, were applied to vary $D_{O2}$ as stated in Table 1. A variation of the Na_2_SO_3_ concentration leads to a change in the liquid viscosity which results in a change in the Galilei number and Schmidt number in Eq. 1. Thus, the influence of the Galilei number was considered during the determination of the exponent ϵ of the Schmidt number (Figure 6). The average value of ϵ = -0.12 can only be considered as a rough estimation because of the high experimental deviation for ϵ between values of −0.227 and 0.022 and the restricted range of variation for the Schmidt number between 417 and 878. However, the exponents of the Galilei number and Schmidt number are notably smaller than the exponents of the remaining numbers which implies that the influence of inaccuracies in these exponents on the k_L_a number is also notably smaller.

**Table 1:** Oxygen solubility, diffusion coefficient and kinematic viscosity for the applied solutions at 25°C

**Composition of the solution**	**Solubility (L**_**O2**_**) [mol/(m**^**3**^**∙bar)]**	**Diffusion coefficient for oxygen (**$D_{O2}$**) [m**^**2**^**/s]**	**Kinematic viscosity (ν) [m**^**2**^**/s]**
Deionized water	1.227	2.14 · 10^-9^	0.89 · 10^-6^
0.5 mol/L Na_2_SO_3_	0.844	1.897 · 10^-9^	1.10 · 10^-6^
1 · 10^-7^ mol/L CoSO_4_			
0.012 mol/L phosphate buffer (pH 8)			
1 mol/L Na_2_SO_3_	0.561	1.688 · 10^-9^	1.48 · 10^-6^
1 · 10^-7^ mol/L CoSO_4_			
0.012 mol/L phosphate buffer (pH 8)			

With the determined exponents, the complete k_L_a correlation can be written as:

(3) $$\begin{array}{l} k_{L}a\cdot {\left(\frac{\nu }{g^{2}}\right)}^{\frac{1}{3}}=1.06\cdot 10^{‒3}\cdot {\left(\frac{n^{2}\cdot d_{0}}{g}\right)}^{1.06}\cdot {\left(\frac{V_{L}}{d^{3}}\right)}^{‒1.2}\\ \;\cdot {\left(\frac{d_{0}}{d}\right)}^{‒1.06}\cdot {\left(\frac{d^{3}\cdot g}{\nu^{2}}\right)}^{‒0.12}\cdot {\left(\frac{\nu }{D_{O2}}\right)}^{‒0.12} \end{array}$$

The constant coefficient of 1.06∙10^-3^ in Eq. 3 was fitted by comparing all measured and calculated values using the method of least squares. Equation 3 can be simplified to the following final k_L_a correlation:

(4) $$k_{L}a=1.06\cdot 10^{‒3}\cdot d^{4.3}\cdot n^{2.12}\cdot {V_{L}}^{‒1.2}\cdot \nu^{‒0.21}\cdot {D_{O2}}^{0.12}\cdot g^{‒0.51}$$

The same exponents for the Froude number and the Geometric number in Eq. 3 led to an elimination of the shaking diameter d_0_ in Eq. 4. Equations 3 and 4 are only applicable for shaking frequencies that are higher than the critical frequency N_C_ according to Eq. 2 and are restricted to the following variation ranges for the Froude number: 0.013 <$\frac{n^{2}\cdot d_{0}}{g}$ < 0.097, the Volume number: 0.18 <$\frac{V_{L}}{d^{3}}$ < 0.65, the Geometric number: 0.06 <$\frac{d_{0}}{d}$ < 0.42, the Galilei number: 6.2∙10^10^ <$\frac{d^{3}\cdot g}{\nu^{2}}$ < 1.88∙10^12^ and the Schmidt number: 417 <$\frac{\nu }{D_{O2}}$ < 878. Exponents of the reactor diameter d and the shaking frequency n in Eq. 4 are more than double those of respective exponents of correlations for shake flasks as summarized by Klöckner and Büchs [34]. For instance, Henzler and Schedel [26] proposed the following k_L_a correlation for shake flasks:

(5) $$k_{L}a=0.5\cdot d^{2.03}\cdot n\cdot {V_{L}}^{‒0.89}\cdot \nu^{‒0.24}\cdot {D_{O2}}^{0.5}\cdot g^{‒0.13}\cdot d_{0}^{0.25}$$

An exponent of 2.03 for the influence of the reactor diameter d and 1 for the influence of the shaking frequency n were specified in Eq. 5 for shake flasks. Different characteristics with respect to the oxygen transfer are based on differences in the shape of the reactor systems. The conical shape of the shake flask wall prevents a strong expansion of the liquid surface with increasing shaking frequency and vessel size. This is not the case in cylindrical orbitally shaken bioreactors, resulting in higher exponents for the corresponding variables n and d.

**Figure 7:**
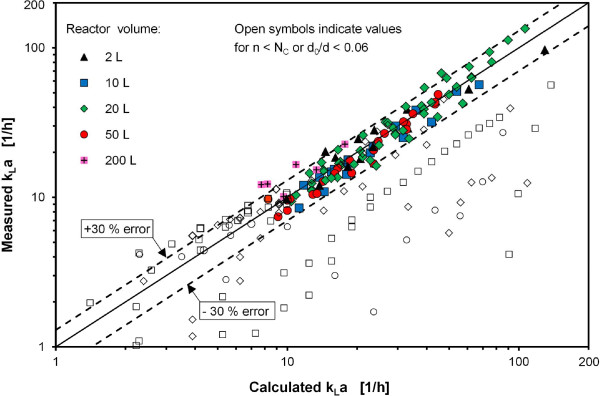
**Comparison between measured and calculated k**_**L**_**a values at 25°C for Corning roller bottles (2 L), Nalgene vessels (10 L, 20 L, 50 L) and CultiBag ORB (200 L) bioreactors.** Shaking frequencies of between 60 rpm and 220 rpm and relative filling volumes between 25% and 75%. Open symbols indicate values for n < N_C_ or d_0_/d <0.06. Values measured with water and sulfite solutions with 0.5 M and 1 M Na_2_SO_3,_ 10^-7^ M CoSO_4_, 0.012 M phosphate buffer and initial pH = 8.

Experimentally determined k_L_a values in this study were compared with calculated values using Eq. 4. A comparison between measured and calculated k_L_a values in scales from 2 L to 200 L is presented in Figure 7. Filled symbols indicate measuring values where the shaking frequency was higher than the critical frequency N_C_ according to Eq. 2. A range for the accuracy of +/− 30% was determined for bioreactors with volumes from 2 L to 50 L as depicted in Figure 7. Open symbols that fall within the +/− 30% accuracy range are still in-phase but close to out-of-phase operation. It was necessary to exclude these data points to define a precise validity range for the correlation. Three measurement values of the 200 L reactor system were slightly above the 30% range (Figure 7). This deviation might be caused by the oxygen-transfer enhancing influence of welded plastic seams in the 200 L bag system. This effect was not considered in Eq. 4, because most of the measurements for determining the exponents in Eq. 4 were conducted with disposable systems with volumes ranging from 2 L to 50 L that provide a smooth and homogeneous surface of the inner reactor wall.

The application of dimensionless numbers requires geometrical similarity. The utilized reactor systems for determining the exponents in Eq. 4 had a cylindrical reactor wall with flat bottom geometry. Thus, Eq. 4 is only applicable for such cylindrical bioreactors. The developed k_L_a correlation can be used to calculate suitable shaking parameters for a sufficient oxygen supply by calculating the maximum oxygen transfer capacity (OTR_max_) according to Eq. 10. To avoid an oxygen limitation during scale-up it is essential to select shaking parameters where the OTR_max_ is higher than the oxygen uptake of the culture.

The newly developed k_L_a correlation was used to determine suitable cultivation conditions for a sufficient oxygen supply of *Nicotiana tabacum* cv. Bright Yellow-2 (BY-2) suspension cells. A maximum OTR value of 6–8 mmol/(L∙h) was measured during the cultivation of tobacco BY-2 cells in 250 mL shake flasks using Murashige and Skoog medium supplemented with 30 g/L sucrose [35]. Thus, a maximum oxygen transfer capacity of OTR_max_ = 8 mmol/(L∙h) is required to ensure a sufficient oxygen supply during cultivation of BY-2 cells in orbitally shaken bioreactors. As specified with Eq. 10, the OTR_max_ is a function of the k_L_a, the dissolved oxygen concentration at equilibrium conditions ($L_{O_{2}}$) and the oxygen partial pressure in the gas phase ($p_{O_{2}}$). The dissolved oxygen concentration at equilibrium conditions in Murashige and Skoog medium supplemented with 30 g/L sucrose at ambient air pressure and 25°C was specified by Curtis [36] as: $L_{O_{2}}\cdot p_{O_{2}}$ = 7.92 mg/L : 32 g/mol = 0.248 mmol/L. For the minimum required k_L_a value it follows:

(6) $$k_{L}a=\frac{{\mathrm{OTR}}_{\max}}{L_{O_{2}}\cdot p_{O_{2}}}=\frac{8\;\mathrm{mmol}/\left(L\cdot h\right)}{\;0.248\;\mathrm{mmol}/L}=32\;\frac{1}{h}$$

Consequently, during scale up from shake flasks to orbitally shaken bioreactors a minimum k_L_a value of 32 1/h is required to avoid an oxygen limitation of tobacco BY-2 cells cultivated in Murashige and Skoog medium containing 30 g/L sucrose. The required shaking parameters were determined according to Eq. (4) with a measured kinematic viscosity of ν = 1.18 ∙ 10^−6^ m^2^/s, a diffusion coefficient for oxygen of D_O2_ = 1.72 ∙ 10^−9^ m^2^/s estimated according to Jamnongwong et al. [37] for Murashige and Skoog medium and the gravitational acceleration of g = 9.81 m/s^2^. The required shaking frequency for a 10 L reactor system (d = 0.24 m) with a filling volume of V_L_ = 3 L results from Eq. (4):

(7) $$n={\left(\frac{k_{L}a}{1.06\cdot 10^{‒3}\cdot d^{4.3}\cdot {V_{L}}^{‒1.2}\cdot \nu^{‒0.21}\cdot {D_{O2}}^{0.12}\cdot g^{‒0.51}}\right)}^{\frac{1}{2.12}}=156\cdot \mathrm{rpm}$$

**Figure 8:**
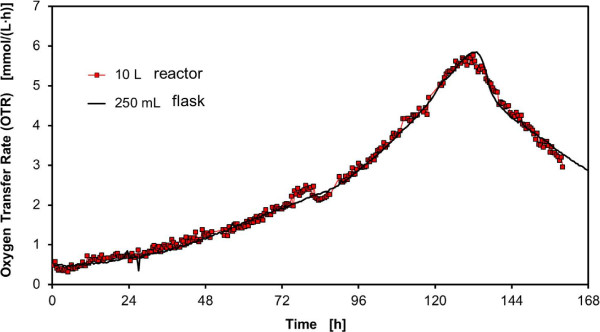
**Oxygen Transfer Rate (OTR) during the cultivation of *N. tabacum*BY-2 MTAD cells cultivated in Murashige and Skoog medium in a 10 L bioreactor, V**_**L**_ **= 3 L, n = 160 rpm, d**_**0**_ **= 7 cm and 250 mL shake flask, V**_**L**_ **= 50 mL, n = 180 rpm, d**_**0**_ **= 5 cm.**

A minimum shaking frequency of n = 156 rpm is required according to Eq. (7) to avoid an oxygen limitation at the aforementioned conditions. Figure 8 shows a comparison of the OTR signals of BY-2 cells cultivated in a 10 L orbitally shaken bioreactor and a 250 mL RAMOS shake flask.

A similar trend of the OTR signal was achieved at a shaking frequency of n = 160 rpm in the 10 L reactor system compared to the 250 mL shake flask. A plateau of the OTR signal, as it usually appears during cultivations with oxygen limitation [38], was not observed.

**Figure 9:**
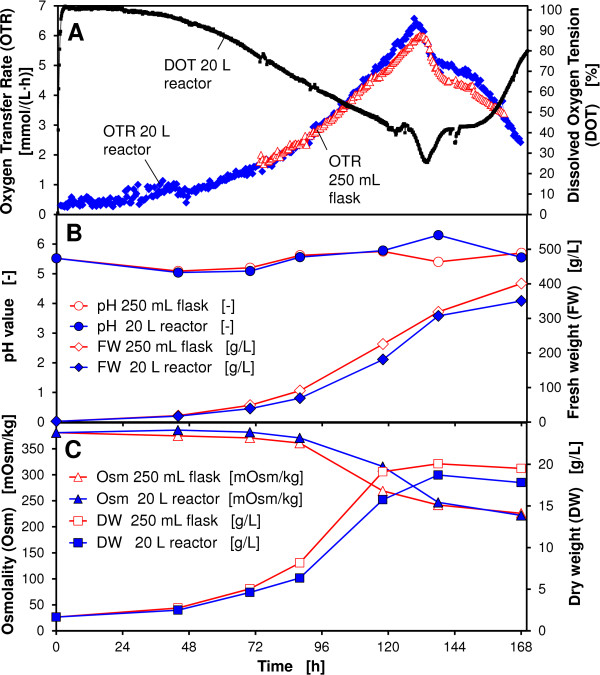
**Comparison of oxygen transfer and growth of *N. tabacum *BY-2 MTAD cells cultivated in KNO**_**3 **_**enriched Murashige and Skoog medium; 20 L bioreactor, V**_**L**_ **= 5 L, n = 160 rpm, d**_**0**_ **= 7 cm and 250 mL shake flasks, V**_**L**_ **= 50 mL, n = 180 rpm, d**_**0**_ **= 5 cm.**

**Figure 10:**
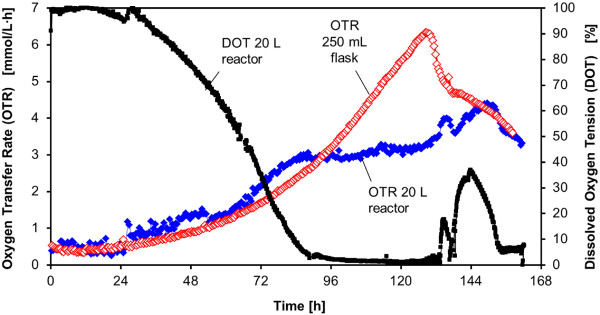
**Oxygen Transfer Rate (OTR) and Dissolved Oxygen Tension (DOT) during the cultivation of *N. tabacum *BY-2 MTAD cells in KNO**_**3 **_**enriched Murashige and Skoog medium in a 20 L reactor, V**_**L**_ **= 10 L, n = 160 rpm, d**_**0**_ **= 7 cm and 250 mL shake flask, V**_**L**_ **= 50 mL, n = 180 rpm, d**_**0**_ **= 5 cm.**

The minimum required shaking frequency for the cultivation of BY-2 cells in a 20 L reactor system (d = 0.286) with a filling volume of V_L_ = 5 L is determined analogously to Eq. (7) to: n = 146 rpm. Thus, a shaking frequency of n = 160 rpm leads to a sufficient oxygen transfer during the cultivation of BY-2 cells. Figure 9 shows a comparison of the oxygen transfer and growth parameters during the cultivation of BY-2 cells in a 20 L bioreactor system compared to a 250 mL shake flask culture. The dissolved oxygen tension (DOT) was additionally monitored in the 20 L bioreactor system (Figure 9). The DOT signal was always above 20% during the cultivation, indicating a sufficient oxygen supply. As depicted in Figure 9, a comparable trend of growth parameters between the 20 L bioreactor system and 250 mL shake flasks was observed. An increase of the filling volume in the 20 L reactor system from 5 L to 10 L at otherwise equal conditions leads to a reduced k_L_a value according to Eq. (7) of k_L_a =17 1/h. This value is significantly lower than the required k_L_a value of 32 1/h for the cultivation of BY-2 cells. Consequently, a filling volume of 10 L leads to an oxygen limitation at the specified conditions as shown in Figure 10. The oxygen limitation is indicated by the plateau of the OTR signal after a cultivation time of 88 h. At the same time the dissolved oxygen tension was close to zero as an additional proof for an oxygen limitation. The results demonstrated that the developed k_L_a correlation can be used to determine suitable cultivation conditions for a sufficient oxygen transfer at different scales.

## Conclusions

A universally applicable equation was defined for calculating the mass transfer coefficient k_L_a in disposable cylindrical bioreactors with volumes ranging from 2 L to 200 L. Important parameters as the critical circulation frequency for the induction of liquid motion as well as in-phase operation conditions were considered during the experiments and the data evaluation. It was demonstrated that the final k_L_a equation can be applied to determine the OTR_max_ of cylindrical bioreactors and, in this way, enables the identification of suitable cultivation conditions such as for plant cell suspension cultures. As a result, the derived k_L_a equation is an essential tool for the correct application of cylindrical disposable shaken bioreactors on a bench- to pilot-scale.

## Materials and methods

### Cultivation and agitation systems

Table 2 presents an overview of the different geometries and material properties of the utilized disposable reactor systems and the respective shaking platform used in this study.

**Table 2:** Properties of the utilized disposable reactor systems

**Product name**	**Nominal volume**	**Reactor diameter (d)**	**Material**	**Shaking platform**	**N**_**c**_**for V**_**L**_ **= 20% (Eq.**2**)**
Corning roller bottle, 850 cm^2^, easy grip vent cap	2 L	116.3 mm	PS^b^	Kühner ISF1-X	147 rpm
Nalgene 10 L Clearboy	10 L	240 mm	PC^c^	Kühner SR 200-X Pilot-Shaker	77 rpm
Nalgene 20 L Carboy	20 L	286 mm	PP^a^	Kühner SR 200-X Pilot-Shaker	76 rpm
Nalgene 50 L Carboy	50 L	379 mm	PP^a^	Kühner SR 200-X Pilot-Shaker	69 rpm
Sartorius 200 L CultiBag ORB	200 L	750 mm	LDPE^d^/EVOH^e^/EVA^f^	Kühner SB 200-X OrbShake	35 rpm

^a^ PP: Polypropylene, ^b^ PS: Polystyrene, ^c^ PC: Polycarbonate, ^d^ LDPE: Low density polyethylene, ^e^ EVOH: Ethyl vinyl alcohol, ^f^ EVA: Ethylene-vinyl acetate.

### Model for the gas/liquid oxygen transfer

The oxygen transfer rate (OTR) from the gas phase to the liquid phase is commonly described as a function of the volumetric mass transfer coefficient (k_L_a) and the concentration difference between oxygen at the saturated gas/liquid interface $\left(C_{L}^{*}\right)$ and oxygen in the bulk phase (C_L_) as:

(8) $$\mathrm{OTR}=k_{L}a\cdot \left(C_{L}^{*}-C_{L}\right)$$

The oxygen concentration $C_{L}^{*}$ is a function of the oxygen solubility $\left(L_{O_{2}}\right)$ and the oxygen partial pressure in the gas phase $\left(p_{O_{2}}\right)$ With Eq. 8, it follows:

(9) $$\mathrm{OTR}=k_{L}a\cdot \left(L_{O_{2}}\cdot p_{O_{2}}-C_{L}\right)$$

The oxygen solubility $\left(L_{O_{2}}\right)$ depends on the concentrations of salts and organic compounds in the cultivation media and can be estimated for salt solutions according to Weisenberger and Schumpe [39] or for aqueous solutions of organic compounds according to Rischbieter et al. [40]. The influence of media components on the oxygen solubility, as discussed by Curtis for plant cell cultivation media [36], depends on the specific media composition. The maximum oxygen transfer capacity (OTR_max_) is reached at a dissolved oxygen concentration in the liquid bulk phase close to zero (C_L_ ≈ 0 mol/L) [28,41]. For the OTR_max_ follows from Eq. 9:

(10) $${\mathrm{OTR}}_{\max}=k_{L}a\cdot L_{O_{2}}\cdot p_{O_{2}}$$

Equation 10 can be rewritten as:

(11) $$k_{L}a=\frac{{\mathrm{OTR}}_{\max}}{p_{O_{2}}\cdot L_{O_{2}}}$$

Values for the mass transfer coefficient k_L_a were calculated from the measured OTR_max_ signal according to Eq. 11.

### The sulfite reaction system

A sodium sulfite (Na_2_SO_3_) reaction can be used to reduce the dissolved oxygen in the bulk phase (C_L_), and, in this way, to simulate oxygen consumption by a biological culture [28,42-44]. The main advantages of a chemical reaction compared to a biological culture are the constant and reproducible oxygen consumption without sterility problems. The cobalt catalyzed sulfite oxidation is described by the following stoichiometric equation [44]:

(12) $${\mathrm{SO}}_{3}^{2-}+1/2\cdot O_{2}\overset{{\mathrm{Co}}^{2+}}{\to }{\mathrm{SO}}_{4}^{2-}$$

Different reaction rates of the sodium sulfite oxidation reaction can be adjusted by varying the cobalt concentration. A non-accelerated reaction rate with a Hatta number (Ha) of Ha < 0.3 is required for k_L_a measurements [44]. In this range, the sulfite reaction is able to reduce the dissolved oxygen concentration (C_L_) to values close to zero. An increase in the oxygen transfer rate leads to a slight increase in C_L_ as described by Maier et al. [42]. Thus, C_L_ needs to be considered during measurements with high oxygen transfer rates, as they are usually reached in bubble aerated stirred tank reactors [42]. As comparatively low oxygen transfer rates of less than 16 mmol/(L∙h) were achieved with the disposable reactor systems in the present work, the assumption of (C_L_ ≈ 0 mol/L) is, in this case, applicable for the determination of k_L_a values.

Sodium sulfite (Roth, Karlsruhe, Germany, purity < 98%) dissolved in deionized water and catalyzed with cobalt sulfate (Fluka, Neu-Ulm, Germany) was used for the oxidation reaction in the liquid phase. Two different sulfite concentrations of 0.5 mol/L and 1 mol/L were used to vary the diffusion coefficient for oxygen $\left(D_{02}\right)$ in the liquid phase. Both solutions were buffered with 12 mmol Na_2_HPO_4_/NaH_2_PO_4_ buffer, and a pH value of 8 was adjusted with 30% (w/w) sulfuric acid. The oxygen solubility of the solutions were calculated according to Weisenberger and Schumpe [39] and oxygen diffusion coefficients according to Akita [45]. A ratio of $D_{O2,\mathrm{Sulfite}}/D_{O2,\mathrm{Water}}$ = 0.886 was determined for the 0.5 mol/L sulfite solution and a ratio of $D_{O2,\mathrm{Sulfite}}/D_{O2,\mathrm{Water}}$ = 0.788 was found for the 1 mol/L sulfite solution in agreement with values calculated by Linek and Vacek [46] using the same model proposed by Akita [45]. The viscosity was measured with an Anton Paar MCR 301 rheometer (Anton Paar GmbH, Graz, Austria). Values for the oxygen solubility, diffusion coefficients and liquid viscosity are listed in Table 1.

### Adaption of the Respiration Activity Monitoring System (RAMOS)

**Figure 11:**
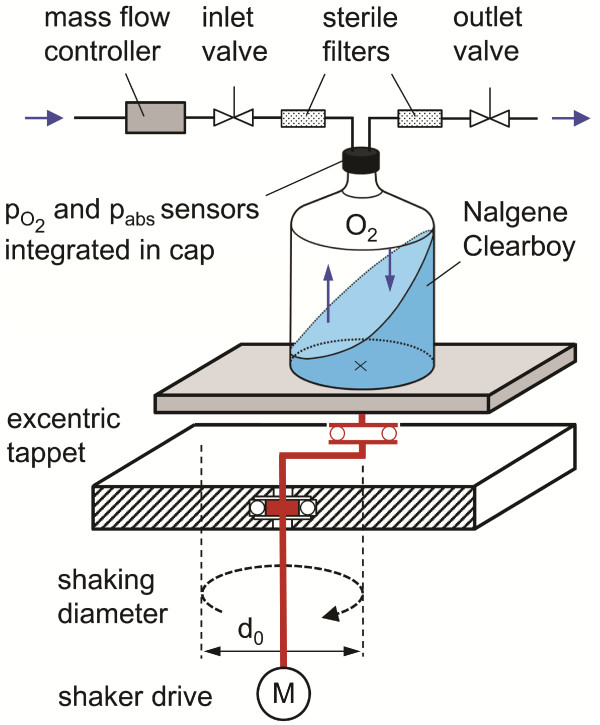
**Experimental setup of the RAMOS for determining k**_**L**_**a values in cylindrical disposable bioreactors with sensors for the oxygen partial pressure (p**_**O2**_**) and total air pressure (p**_**abs**_**) integrated in the reactor cap; mass flow controller, inlet and outlet valves and sterile filters used for active aeration.**

The Respiration Activity Monitoring System (RAMOS), initially developed for shake flasks, was adapted to the different disposable bioreactor systems with volumes ranging from 2 L to 50 L to measure the OTR_max_ in combination with the sulfite reaction system. Values in the 200 L bioreactor system were determined with the dynamic gassing out method. A detailed description of the RAMOS device for shake flasks is given by Anderlei and Büchs [38] and Anderlei et al. [47]. A scheme of the modified RAMOS for cylindrical reactors is shown in Figure 11. An electrochemical oxygen partial pressure sensor (MAX-250B, Maxtec, Salt Lake City, UT, USA) and a total pressure sensor (No. 26PCAFA6D, Honeywell Sensing and Control, Golden Valley, MN, USA) were integrated in the headspace of the reactor systems. Magnetic flipper valves (Type 0332 E, Bürkert, Ingelfingen, Germany) were used to switch between measuring and rinsing phase and a mass flow controller was used to control the aeration rate during the rinsing phase (see section about aeration). Otherwise, the setup of the RAMOS device and the OTR calculation were like those for shake flasks described by Anderlei and Büchs [38]. Measurement values for OTR_max_ were used to determined k_L_a values according to Eq. 11.

### Aeration of the 2 L Corning roller bottle reactor

The Corning roller bottle reactor (Corning Inc., Acton, MA, USA) with vent cap is equipped with a 0.2 μm membrane that is integrated in the cap to allow a sufficient aeration during cultivation. A method developed by Anderlei et al. [48] for determining the mass transfer resistance of shake flask closures was applied to measure the diffusive mass transfer through the membrane of the cultivation system. A volume flow rate of 9 mL/min was determined with open ports at 25°C. This value was used for the active aeration with a Brooks 5850 TR mass flow controller (Brooks Instrument, Ede, The Netherlands) in the RAMOS as pictured in Figure 11. An oxygen sensor was placed on top of the reactor systems to measure the oxygen partial pressure in the gas phase. No further changes of the RAMOS for shake flasks were required to measure the OTR_max_ of the Corning roller bottle cultivation system.

### Aeration of cylindrical orbitally shaken bioreactors with volumes from 10 L to 200 L

The settings of the air flow rate for the active headspace aeration of the cylindrical shaken bioreactors was transferred from the RAMOS device for shake flasks and adjusted according to the nominal reactor volume. A volume flow rate of 10 mL/min is usually applied in the RAMOS device for 250 mL shake flasks to mimic the oxygen transfer through a conventional cotton plug of shake flasks with a narrow neck. Consequently, an air flow rate of 400 mL/min was used for a volume of 10 L, 800 mL/min for 20 L, 2 L/min for 50 L and 8 L/min for 200 L bioreactors, respectively. For reactor volumes from 2–50 L, the flow rate was adjusted with a Brooks 5850-E mass flow controller (Brooks Instrument) in the RAMOS as illustrated in Figure 11. A manual air flow meter was used for the dynamic gassing-out method in the 200 L reactor system. The air flow rates in different scales were high enough to keep the absolute headspace concentration of oxygen, measured with the oxygen partial pressure sensor, above 20% during all OTR measurements.

### Application of the dynamic gassing-out method for k_L_a measurements with water

A defined and constant headspace volume is required for OTR_max_ measurements with RAMOS [38]. This could not be provided in the flexible 200 L bag reactor system. Thus, the dynamic gassing-out method, first described by Bandyopadhyay et al. [49], was used for k_L_a measurements in the 200 L scale. Nitrogen was used to replace the dissolved oxygen in the liquid phase, and deionized water was used as medium. Oxygen-sensitive spots (type SP-PSt3-YAU-D5-YOP) from PreSens (PreSens GmbH, Regensburg, Germany) were applied to measure the dissolved oxygen tension (DOT) in the liquid phase. An electrochemical sensor (MAX-250B, Maxtec, Salt Lake City, UT, USA) was additionally used to measure the oxygen concentration in the headspace of the reactor. The reactor system was filled with deionized water according to the desired filling volume. Then, the headspace of the system was filled with nitrogen, and the reactor was shaken at 80 rpm until the DOT reached a value below 1%. Subsequently, the shaker was stopped, and the gas volume in the headspace was replaced with air until the relative oxygen concentration in the headspace reached a value above 98%. The shaker was then immediately started with the designated shaking frequency, and the DOT was recorded over time. During the required time to replace the nitrogen in the headspace with air, the diffusion of oxygen from the gas phase to the liquid phase could cause small oxygen concentration differences in the liquid phase. Therefore, measured DOT values during the first 60 s were not considered for the calculation of the mass transfer coefficient (k_L_a) to ensure a sufficient mixing of the liquid phase. According to Tissot et al. [16], a mixing time of 60 s can be regarded as sufficient for a 200 L reactor system using liquids with water-like viscosity and shaking frequencies above 60 rpm. During all measurements the DOT signal after the 60 s mixing step was below 4%, indicating that only small amounts of oxygen were transferred to the liquid phase during the time needed to replace the gas in the headspace. Values for k_L_a were calculated from the recorded DOT signal over time as described by Van Suijdam et al. [50] and recently summarized by Suresh et al. [51].

### Cultivation of *Nicotiana tabacum* cv. BY-2 plant cell suspension cultures

For evaluation of the k_L_a correlation the transgenic *N. tabacum* cv. Bright Yellow-2 (BY-2) MTAD cell line producing the human antibody M12 was used. The generation of the transgenic BY-2 cell line is described by Raven et al. [52]. BY-2 plant cell suspension cultures were cultivated at 26°C in the dark using Murashige and Skoog media [53] with minimal organics (# M6899, Sigma Aldrich, Saint Louis, MO, USA) supplemented with 30 g/L sucrose, 0.2 g/L KH_2_PO_4_, 0.6 mg/L thiamine-HCl, 0.2 mg/L 2.4-Dichlorophenoxyacetic acid (2.4-D) and, where stated, additional 100 mmol/L KNO_3_. The pH of the culture medium was adjusted to 5.8 with 1 mol/L KOH before autoclaving for 21 min at 121°C. Plant cell suspensions cultures were sub-cultivated weekly for cell maintenance in 250 mL Erlenmeyer flasks, filled with 50 ml cell suspension, sealed with a cotton plug and shaken at 180 rpm and 26°C in the dark. Inoculation was conducted by adding 5% (V/V) of a seven day old culture to fresh medium.

### Oxygen transfer rate measurements in shake flasks

The OTR signals in 250 mL Erlenmeyer flasks were measured with a Respiration Activity Monitoring System (RAMOS). A detailed description of the device and its applications is provided by Anderlei et al. 2004 [47] and Anderlei and Büchs 2001 [38]. The non-invasive measuring system allows online monitoring of the OTR, CTR and RQ without changing the culture conditions compared to conventional 250 mL shake flasks [47]. Conventional Erlenmeyer flasks were used in addition to the RAMOS flasks to take samples during the cultivations.

### Determination of fresh and dry cell weight

An electronic precision balance (SBC 31, Scaltec, Göttingen, Germany) was used to determine fresh and dry cell weight. Fresh cell weight was determined by vacuum filtration of 10 ml cell suspension for 3 min using Whatman filter paper grade 3 (# 1003–055, 55 mm diameter, Fisher Scientific GmbH, Schwerte, Germany). Prior to the filtration step the filter paper was weighted dry and after wetting with purified water. The fresh cell weight was determined from the difference in weight of the membrane with cells directly after filtration and the wet membrane without cells. The membrane with cells was dried at 105°C until the mass remained constant. The dry cell weight was determined from the difference in weight of the dried membrane with and without cells.

### Determination of osmolality, dissolved oxygen tension and pH

Osmolality was determined in the supernatant with a Gonotec Osmomat 030 (Gonotec GmbH, Berlin, Germany). The device was calibrated with a two point calibration prior to each measurement. The pH value was determined with a pH510 pH meter (Eutech, Fisher Scientific GmbH, Schwerte, Germany). The dissolved oxygen tension (DOT) was measured by using oxygen sensitive sensor spots (type SP-PSt3-YAU-D5-YOP) from PreSens (PreSens GmbH, Regensburg, Germany).

## Competing interests

The authors declare that they have no competing interests.

## Authors’ contributions

WK conducted experiments, made the data evaluation and drafted the manuscript. RG and TA provided additional data and helped to conduct the experiments. NR provided plant cell suspension cultures, helped to conduct the experiments and reviewed the manuscript. SS coordinated the experiments and reviewed the manuscript. CL calculated oxygen diffusion coefficients and solubilities of the applied solutions. JB helped to design and coordinate the study and reviewed the manuscript. All authors read and approved the final manuscript.
